# Dr. Carter's Hospital Reports

**Published:** 1826-07

**Authors:** 


### Hospital Reports.*

Dr. Carter continues his reports in a most candid manner, by which he, at once, renders them useful, and gives them the best voucher for authenticity.—We shall extract a few particulars from his last report.

1. *Epilepsy.* Five cases of this complaint are given, from which it would appear that tartar emetic externally applied has been a very efficacious remedy.

*Case* 1. A lad, aged 14 years, had been affected with epileptic fits for ten years, and averred that, during that time, he had never been a fortnight free from them. Nothing was ordered but the tartar emetic ointment to be rubbed on the left arm, with a common laxative occasionally. The eruption was kept up, more or less, for nearly three months, and he never had a fit or any threatening of one. Discharged cured.

*Case* 2. This was also a lad of 15, who had had epilepsy for seven years, the fits latterly becoming very frequent, and sometimes recurring several times a day. He used the ointment, and took ol. terebinth. thrice a day, in doses of 25 drops. He was not cured, but the fits were rendered so bearable, that he was able to pursue his usual avocations.

*Case* 3. This was a young female, aged 16 years. The disease was recent. She had been bled and purged freely, but without much benefit. Her appearance was plethoric, yet the circulation was very languid, extremities cold, bowels costive. She was ordered a dose of calomel and antimonial powder, after which the ointment was to be applied to the left arm, and she was to take Griffith's mixture. For nearly two months, during which the sore on the arm was open, she was almost free from fits; but no sooner was the sore closed than the epilepsy returned. The ointment and steel medicine were again renewed, and she had no fit for three months, when she was discharged.

---

* Dr. Carter, Physician to the Kent and Canterbury Hospital.—Repos. April, 1826.

*Case* 4. A labourer had had sub-acute hepatitis, which was removed by local and general bleeding, mercurials, and saline purgatives. After this, he became affected with epileptic fits, to which he had not been previously subject. Tartar emetic ointment was applied to the arm, and leeches to the temples. The epileptic fits did not return.

*Case* 5. A woman, aged 21 years, was admitted on the 14th January, the fits recurring at uncertain intervals, apparently in consequence of a blow she had received on the nucha, about a year previously. The longest interval was eleven weeks. She had an epileptic fit in the hospital on the 22d January. On examination, some tenderness was perceptible in the site of the blow. Leeches were, therefore, applied, and aperients were prescribed. The antimonial ointment was applied to the shaven occiput. She appears to have been cured.

The remedy in question deserves, at all events, to be employed as an *auxiliary*—we fear it will not often be found to permanently cure the complaint, as the *principal.*

II. *Bronchocele.* Four cases of this complaint are detailed by Dr. Carter. In the first case, the disease was of two years standing, in a female, and the circumference of the neck was 16 inches, the tumour being hard, the respiration impeded and the voice altered—general health good. She used the iodine ointment for a month, and took six drops of the tincture thrice a day, without any impression being made on the tumour. A blister was then applied, and the dose increased to ten drops. She also took a mercurial pill occasionally. The ointment was again employed in increased quantity. The tumour became stationary, whereas it had previously been gradually augmenting. The dimensions of the throat were reduced $2\frac{1}{2}$ inches, and the tumour was rendered softer; but no farther impression could be made on the complaint.

*Case* 2. A married female, aged 31. The bronchocele had existed for several years, but latterly it had increased much. The throat, on admission, measured $15\frac{1}{2}$ inches. She was in bad health. The iodine ointment was used for a good while; but the tincture could not be borne beyond a week. Leeches, blisters, and other means were also used. At the expiration of two months there was scarcely any impression made on the bronchocele; but in the course of the succeeding two months, the tumour was sensibly diminished, and ultimately the circumference of the neck was reduced 1 inch and $\frac{3}{4}$. There was perceptible wasting of the mamma in this case, during the use of the iodine ointment.

*Case* 3. A girl, ten years of age, had a neck 12 inches in circumference. In 27 days, during which she used the ointment, there was a diminution of one inch. The bronchocele ultimately vanished entirely under the remedy.

*Case* 4. A girl aged 17, had bronchocele for some years. The tumour had increased rapidly since she attained the age of puberty. Circumference of the neck 18 inches—great fulness and pain of the head—troublesome dyspepsia—difficult respiration. The iodine ointment produced no effect till after the application of leeches and a blister; then the ointment began to tell on the disease, and the tumour was ultimately considerably reduced in size. She was discharged not quite cured; but greatly relieved.

III. *Paralysis from Cold.* A lad had been exposed to wet and cold, and in that state sat in his wet clothes. This was succeeded by complete paralysis of the lower extremities, difficult micturition, and torpid bowels. No fall nor other accident had occurred, nor could any affection of the spine be detected. Leeches, purgatives, and stimulating liniments. Half a drachm of oil of turpentine was also ordered thrice a day. These means were reiterated, but no impression was made on the complaint. Caustic issues were then inserted, and the medicines continued. No sooner had the issues begun to discharge, than a manifest improvement took place, and continued progressive till the use of the lower extremities was completely recovered.

IV. *Obstinate Vomiting.* This was a curious case, on more accounts than one. The patient was a female, aged 24 years, who applied on the 26th August, 1824, complaining of vomiting her food soon after it was taken, attended with burning pain at the pit of the stomach and between the shoulders. This state had continued for two years. The pulse was frequent and feeble, tongue clean, bowels constipated, catamenia regular. There was tenderness about the scrobiculus cordis, anxious countenance, palpitation—considerable and progressive emaciation. She had been bled, blistered, and had taken various remedies, but without effect, and Dr. Carter also prescribed different medicines without making any impression on the complaint. He then tried an old remedy, the extract of calendula or marigold, in doses of three grains every three hours, all other medicines being laid aside. This produced almost immediate cessation of the vomiting. Pain, however, continuing in the region of the stomach, leeches were, from time to time, applied, and the bowels kept moderately open. The patient gradually improved, and gained flesh and strength. She was ultimately discharged cured.

Dr. C. had his attention directed to this medicine from a notice in a foreign journal, where its efficacy was stated in scirrhus and cancer. Upon giving the medicine, the vomiting ceased, and did not return for three months, the time she was under observation. Should any one be inclined to attribute the cure to the leaving off all other remedies, Dr. C. observes that this was done before, but without the same effect. The calendula is a medicine of ancient, though of almost forgotten, reputation. It seems to deserve a trial in chronic affections of the stomach. In a case of organic disease of the uterus, our author exhibited the calendula, with the effect of mitigating pain and lessening the discharge.

The preparation was an aqueous extract of the flowers, and was furnished by Mr. Battley. About a pound of the flowers produced an ounce of the extract.

V. *Apparent Phthisis.* C. Merit, an upholder, aged 25 years, was admitted on the 19th August, having been ill for some weeks with "strongly marked symptoms of pulmonary consumption." There was also a scrofulous enlargement of one knee. One of his brothers had died of mesenteric consumption. Under such circumstances, the prognosis was unfavourable. An issue was made in the left side—infusion of roses acidulated, and the sulphate of quinine were ordered, with tincture of digitalis. Five grains of the styrax pill were given at bed-time. Leeches were applied occasionally to the chest. By these means the pulse was brought down to its natural standard—the harrassing cough abated—and the expectoration, which had been copious and purulent, became less in quantity and of better quality—in short, before much time had elapsed, "the symptoms of disease of lungs, and the affection of knee were almost entirely gone." But he was annoyed with a troublesome diarrhœa, which obliged our author to discontinue the medicine first prescribed, and to order chalk-mixture with laudanum, &c. He was ultimately discharged apparently cured, and resumed his employments.

We regret that Dr. Carter has omitted to mention the result of auscultation and percussion in this case; as we think there can be little doubt that the disease was confined to the mucous membrane of the lungs, and that the expectoration did not come from the parenchymatous structure.

We thank Dr. Carter for these contributions to practical medicine, and sincerely wish that his example may soon be followed by the medical officers of other similar institutions.

